# Potencial Terapêutico da Vortioxetina: Respostas Cardíacas ao Estresse Crônico Moderado Imprevisível em um Modelo de Rato

**DOI:** 10.36660/abc.20240159

**Published:** 2025-02-05

**Authors:** Ozlem Ozmen, Serife Tasan, Gulin Ozdamar Unal

**Affiliations:** 1 Burdur Mehmet Akif Ersoy University Department of Pathology Burdur Turquia Burdur Mehmet Akif Ersoy University – Department of Pathology, Burdur – Turquia; 2 Suleyman Demirel University Department of Psychiatry Isparta Turquia Suleyman Demirel University – Department of Psychiatry, Isparta – Turquia

**Keywords:** Vortioxetina, Coração, Imuno-Histoquímica, Patologia

## Abstract

**Fundamento:**

O estresse surge em resposta a ameaças ou desafios, afetando a saúde física e mental. Embora seus efeitos nocivos ao coração sejam amplamente reconhecidos, as investigações em nível celular permanecem limitadas. Antidepressivos, incluindo vortioxetina (VOR), são conhecidos por impactar o sistema cardiovascular. VOR, usado para tratar transtorno depressivo maior, é considerado uma opção promissora para pacientes com doença cardíaca devido às suas propriedades anti-inflamatórias e antioxidantes, que podem reduzir danos cardíacos.

**Objetivos:**

Este estudo teve como objetivo avaliar os efeitos do estresse crônico moderado imprevisível (ECMI) em corações de ratos e avaliar os potenciais efeitos protetores do VOR contra danos cardíacos induzidos por estresse.

**Métodos:**

Vinte e oito ratos Wistar Albino machos foram divididos em quatro grupos. O grupo ECMI experimentou estresse diário aleatório por 6 semanas, enquanto o grupo ECMI+VOR recebeu tratamento VOR junto com estresse. Os grupos VOR e controle não foram expostos ao estresse. Amostras de coração foram examinadas histopatologicamente e imuno-histoquimicamente.

**Resultados:**

O grupo ECMI apresentou aumento de hemorragia, edema, degeneração vacuolar e infiltrações de células mononucleares, com redução de troponina e IL-10 e aumento de expressões de caspase-3 e NF-κB em comparação ao grupo controle (p≤0,001). O tratamento com VOR melhorou esses achados, normalizando os resultados histopatológicos e imuno-histoquímicos.

**Conclusões:**

O ECMI causou danos cardíacos significativos em ratos, enquanto o tratamento com VOR mostrou efeitos protetores ao aliviar essas alterações patológicas.

## Graphical abstract

**Figura Central:**
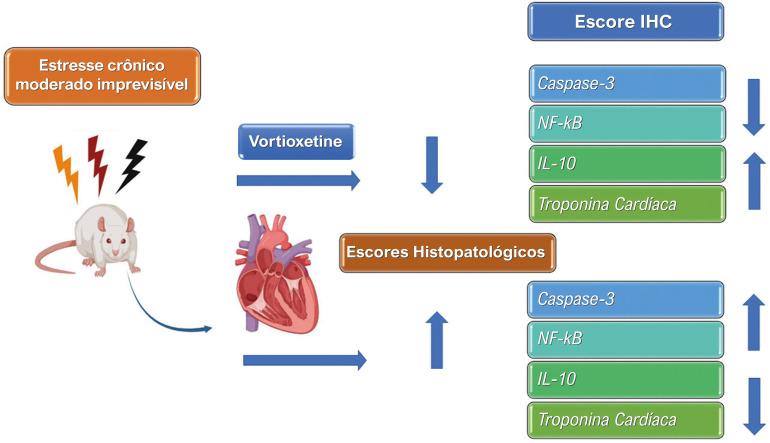
Potencial Terapêutico da Vortioxetina: Respostas Cardíacas ao Estresse Crônico Moderado Imprevisível em um Modelo de Rato Mecanismo patogênico esquemático do dano do ECMI no coração.

## Introdução

O estresse é um problema psicológico significativo e crescente que impacta a vida diária de indivíduos globalmente. Ao mesmo tempo, depressão e ansiedade se destacam como os transtornos psiquiátricos mais prevalentes em muitas sociedades em todo o mundo.^1^ Esses transtornos psicológicos emergem de interações complexas envolvendo fatores neurobiológicos, genéticos e eventos comuns da vida.^2,3^

O cérebro, juntamente com os sistemas cardiovascular e imunológico, constitui alvos comuns de estresse. Pesquisas indicam que fatores psicossociais desempenham um papel fundamental na origem e progressão da doença cardíaca coronária. Quando certas regiões do cérebro são ativadas devido ao estresse, a resposta é gerada através do sistema nervoso simpático ou hormônios, consequentemente influenciando as funções normais do coração. O estresse crônico moderado imprevisível (ECMI) leva a aumentos significativos nos níveis séricos de corticosterona, resultando em um perfil lipídico aterogênico exacerbado que acelera a aterosclerose.^4-6^ Gu et al., em particular, observaram que o ECMI induz alterações morfológicas e fisiológicas que podem contribuir para a aterosclerose.^7^

A ativação do eixo hipotálamo-hipófise-adrenal e o consequente aumento da liberação de glicocorticoides, desencadeados por situações de estresse imprevisíveis e repetitivas, podem resultar em uma produção amplificada de espécies reativas de oxigênio e estresse oxidativo nas células do sistema nervoso central.^4^ O estresse oxidativo desempenha um papel fundamental na patogênese de distúrbios do sistema cardiovascular.^8^

Os modelos ECMI têm sido amplamente utilizados como modelos de depressão em animais experimentais.^9^ Esses modelos replicam estressores ambientais sociais, espelhando de perto o desenvolvimento da depressão em humanos, tornando-os mais adequados para fins investigativos.^10-12^ Comportamentos anedônicos, caracterizados por uma redução na preferência por sacarose em roedores expostos ao estresse, são características-chave deste modelo.^13^ A validade do modelo é ressaltada pela reversibilidade desta redução no efeito hedônico com tratamento crônico envolvendo agentes antidepressivos.^10^

Grippo et al. relataram que ratos expostos a estresse crônico moderado apresentaram aumento da frequência cardíaca, redução da variabilidade da frequência cardíaca, tônus cardíaco simpático elevado, anedonia e diminuição dos níveis de atividade em uma roda de corrida. Suas descobertas sugerem que ratos submetidos a ECMI são vulneráveis a eventos arrítmicos, o que pode levar a resultados cardíacos ainda mais prejudiciais, como infarto do miocárdio ou morte.^14^ Além disso, foi relatado que aumenta a frequência cardíaca devido ao estresse crônico, contribui para a hipertensão e eleva as concentrações de cortisol sérico, sódio e hormônio adrenocorticotrófico.^15^ No entanto, há informações limitadas sobre os achados histológicos e imuno-histoquímicos nos corações de ratos expostos a ECMI.

Os efeitos adversos dos antidepressivos no sistema cardiovascular são conhecidos há muito tempo. Embora os antidepressivos sejam geralmente eficazes na redução da depressão, seu uso em pacientes com doença cardíaca coronária permanece controverso.^16,17^ Portanto, há uma necessidade de explorar antidepressivos que não tenham efeitos prejudiciais no sistema cardiovascular. A vortioxetina (VOR), um antidepressivo multimodal, é empregada no tratamento da depressão e distúrbios cognitivos associados à depressão. O tratamento com VOR tem o potencial de normalizar as mudanças neurocomportamentais induzidas por estressores da vida diária.^18^ No entanto, os mecanismos neurobiológicos que sustentam os efeitos antidepressivos e cognitivos do VOR permanecem incompletamente compreendidos.^19^ Acredita-se que o VOR tenha efeitos positivos no coração devido às suas propriedades anti-inflamatórias e antiapoptóticas.

Além disso, embora o impacto do estresse em doenças coronárias seja bem conhecido, seus efeitos específicos no miocárdio permanecem pouco explorados. Portanto, o objetivo deste estudo é examinar histopatologicamente e imuno-histoquimicamente os efeitos danosos do ECMI no coração, com foco particular na avaliação do papel protetor do VOR na mitigação de lesões cardíacas induzidas por ECMI em um modelo de rato.

## Materiais e métodos

### Animais

Neste estudo, tecidos cardíacos foram obtidos de um projeto originalmente focado na investigação dos efeitos de ECMI no tecido cerebral.^20^ Durante o exame histológico, achados notáveis foram observados nos tecidos cardíacos, o que motivou uma avaliação mais aprofundada com a aprovação do comitê de ética. O estudo foi aprovado pelo Comitê de Ética Local em Pesquisa Animal da Universidade Suleyman Demirel (Aprovação nº: SDU HADYEK 01/02, datado de 06.01.2022). Todos os experimentos foram conduzidos em conformidade com as diretrizes ARRIVE para pesquisa animal.^21^

Usando o software GPower 3.1.9.7, um estudo foi planejado em 4 grupos, cada um consistindo de 7 ratos (tamanho total da amostra de 28), considerando os parâmetros relevantes (α=0,06, 1-β=0,90, tamanho do efeito=0,5). Nesta pesquisa, 28 ratos Wistar Albino machos foram adquiridos do Centro de Pesquisa e Produção Animal Experimental da Universidade Suleyman Demirel, localizado na Universidade Suleyman Demirel, Türkiye. Os ratos tinham 4 semanas de idade, pesavam entre 150-200 gramas e foram confirmados como saudáveis pelo veterinário supervisor na unidade relevante. Os ratos receberam um período de aclimatação de uma semana antes do início do experimento. Após o período de adaptação, eles foram divididos aleatoriamente (atribuição aleatória simples) em quatro grupos, cada um consistindo de sete ratos. O grupo controle não passou por nenhum estresse, enquanto o grupo ECMI passou pelo procedimento de ECMI. O grupo ECMI+VOR foi submetido ao procedimento ECMI e recebeu 10 mg/kg de VOR intraperitonealmente durante as últimas três semanas do experimento. O grupo VOR recebeu 10 mg/kg de VOR intraperitonealmente durante as últimas três semanas sem exposição ao estresse.

Os grupos controle e VOR foram alojados em salas separadas dos grupos ECMI e ECMI+VOR e não foram expostos a nenhum estresse. Os ratos foram alojados em gaiolas Euro-tipo 4 padrão usando aparas de madeira como cama. Todos os ratos foram mantidos sob condições laboratoriais padrão (temperatura: 21°C ± 2°C; umidade: 60% ± 5%; ciclo claro-escuro de 12/12 h) e tiveram acesso ad libitum a uma dieta comercial padrão (Korkuteli Yem, Antalya, Türkiye) e água, exceto durante os períodos de estresse para os grupos ECMI.

### Modelo ECMI

O modelo ECMI foi implementado com base em um método descrito anteriormente com algumas modificações.^22,23^ Os ratos foram alojados individualmente e submetidos a vários estressores como parte do protocolo ECMI ao longo de 6 semanas. Esses estressores incluíram 4 horas em uma gaiola inclinada a 45°, 24 horas de privação de água, 24 horas de jejum, iluminação contínua, 4 horas em uma gaiola com cama molhada, 4 horas de restrição comportamental, 4 horas de estresse social colocando os ratos em gaiolas sujas de outros ratos, 4 horas de estresse hídrico em uma gaiola vazia com 1 cm de água no fundo e 4 horas em uma gaiola vazia. Uma sala dedicada no laboratório foi designada para implementar o protocolo ECMI. Todos os estressores foram aplicados individualmente e continuamente, dia e noite. Os animais de controle foram deixados intactos em suas gaiolas, exceto para manuseio durante a limpeza regular da gaiola.

### Teste de preferência de sacarose

A preferência por sacarose é usada como um indicador de anedonia, um dos principais sintomas da depressão.^24^ Para o teste de preferência por sacarose (TPS), os animais receberam duas garrafas de solução de sacarose 1% p/v e 100 g *ad libitum* no primeiro dia para familiarizá-los com o sabor da sacarose.^25^ No segundo dia, uma das garrafas foi substituída por água. No terceiro dia, os ratos foram privados de comida e água por 23 horas. Uma garrafa continha 100 ml de solução de sacarose 1%, enquanto a outra continha um volume igual de água potável. Os ratos tiveram uma hora para escolher entre os dois líquidos. Para evitar potenciais efeitos de preferência, as posições das garrafas foram trocadas após 30 minutos. A porcentagem de preferência por sacarose foi calculada usando a seguinte fórmula: porcentagem de preferência por sacarose = consumo de sacarose/(consumo de sacarose + consumo de água).^20^

### Tratamento com Vortioxetina

Vortioxetina sintetizada por H. Lundbeck A/S, Istambul, Türkiye, foi dissolvida em água destilada e administrada em uma dose de 10 mg/kg, conforme descrito anteriormente.^26,27^ Os grupos controle e ECMI receberam solução salina estéril a 0,9%. Soluções preparadas recentemente foram injetadas intraperitonealmente em um volume de 1 mL/kg todos os dias no mesmo horário por 3 semanas.^20^ Na conclusão do experimento, todos os ratos foram sacrificados sob anestesia com Xilazina HCl (Xylasinbio %2, Bioveta, República Tcheca) + Ketalar HCl (Ketasol, Richter Pharma AG, Áustria). Posteriormente, amostras foram coletadas para análises histopatológicas e imuno-histoquímicas.

### Método histopatológico

Durante a necropsia, amostras de coração foram coletadas e preservadas em formalina tamponada a 10%. As amostras de tecido passaram por processamento padrão usando equipamento de processamento de tecido totalmente automático e foram embebidas em cera de parafina. Seções de 5 mícrons de espessura foram cortadas dos blocos de parafina. Após a secagem, as preparações foram submetidas a séries de álcool e xileno, coradas com hematoxilina-eosina (HE), montadas com uma lamínula e examinadas sob um microscópio de luz.

Um microscópio de luz com uma objetiva de 40x foi usado para avaliar a gravidade das lesões cardíacas, com fotos tiradas de cinco regiões diferentes do coração de cada animal. As lesões foram classificadas com base em hiperemia, hemorragia, edema, acúmulo de lipídios, degeneração vacuolar, hipereosinofilia no citoplasma das células miocárdicas e infiltrações de células mononucleares. A gravidade das alterações histopatológicas foi avaliada semiquantitativamente como segue: 0, normal; 1, moderado; 2, moderado; e 3, grave.^28^

### Exame imuno-histoquímico

Empregando a técnica de estreptavidina-biotina seguindo as instruções do fabricante, quatro seções consecutivas foram montadas em lâminas revestidas de poli-L-lisina de blocos de parafina previamente preparados e coradas imuno-histoquimicamente. Essas seções foram utilizadas para a detecção de expressões de caspase-3 (Anticorpo anticaspase-3 (E-8): sc-7272, Abcam, Reino Unido), IL-10 (anticorpos IL-10, A16445, Bioscience - EUA), NF-κB (anticorpos anti-NF-κB p65 (ab16502), Abcam, Reino Unido) e troponina cardíaca (anticorpo antitroponina I cardíaca (FNab09781), FineTest, China). Cada anticorpo primário foi aplicado em uma diluição de 1/100, e as seções foram incubadas com esses anticorpos por 60 minutos. A imuno-histoquímica foi realizada usando anticorpos secundários biotinilados e conjugados de estreptavidina-fosfatase alcalina. O anticorpo secundário usado foi o kit EXPOSURE Mouse and Rabbit Specific HRP/DAB Detection IHC (ab80436), e a diaminobenzidina (DAB) serviu como cromógeno (Abcam, Cambridge, Reino Unido). Os controles negativos foram tratados com uma solução de diluição de antígeno em vez do anticorpo primário. Todos os exames foram conduzidos em espécimes cegos por um patologista especializado de outra universidade. Na análise imuno-histoquímica, cada seção foi examinada independentemente para cada anticorpo. Para avaliar a intensidade das reações imuno-histoquímicas em células marcadas por esses anticorpos, uma análise semiquantitativa foi conduzida, empregando uma pontuação de classificação variando de (0) a (3) da seguinte forma: (0) negativo, (1) coloração moderado focal, (2) coloração moderado difusa e (3) coloração marcada difusa. Para avaliação, dez áreas diferentes sob ampliação objetiva de 40X em cada seção foram examinadas. O Database Manual Cell Sens Life Science Imaging Software System (Olympus Co., Tóquio, Japão) foi empregado para análises morfométricas e microfotografia. Os resultados foram registrados e analisados estatisticamente. As análises de pontuação imuno-histoquímica foram realizadas usando o ImageJ versão 1.48 (National Institutes of Health, Bethesda, MD).

### Análise estatística

A análise estatística foi conduzida usando o Statistical Package for Social Sciences (SPSS) 22.00 (SPSS Inc., Chicago, IL, EUA). Inicialmente, a normalidade da distribuição foi avaliada usando o teste de Shapiro-Wilk. Como os dados demonstraram uma distribuição normal (p>0,05), as comparações entre os grupos foram feitas com uma análise de variância unidirecional (ANOVA). O teste de Duncan foi usado para identificar diferenças entre os grupos. Valores de p<0,05 são considerados estatisticamente significativos. O software GraphPad foi usado para os gráficos.

## Resultados

### Achados clínicos

Todos os ratos foram observados diariamente para mudanças comportamentais ao longo do estudo. Foi notado que a porcentagem de preferência por sacarose diminuiu significativamente no grupo ECMI, enquanto os grupos controle, ECMI+VOR e VOR exibiram níveis igualmente altos. Além disso, os ratos no grupo ECMI pareciam mais calmos e mostraram movimentos mais lentos em comparação aos outros grupos. Essas descobertas confirmam a presença de comportamentos depressivos nos ratos submetidos a ECMI.

### Resultados brutos

Na conclusão do período experimental, os animais foram sacrificados de acordo com as diretrizes éticas, e seus corações foram examinados macroscopicamente. Nenhuma alteração patológica grave foi identificada nos corações dos grupos de controle e de estudo durante a necropsia. Os corações de todos os ratos em todo o grupo exibiram aparências macroscópicas normais.

### Achados histopatológicos

**Figura 1:**
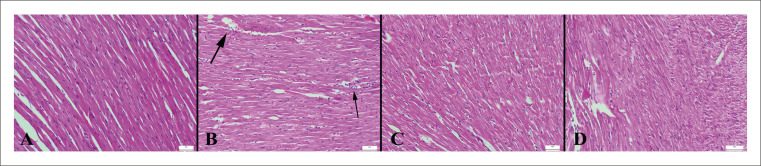
– Aspecto histopatológico dos corações entre os grupos. A) Histologia cardíaca normal no grupo controle; B) Hiperemia, pequenas hemorragias (setas grossas) e infiltrações de células mononucleares (setas finas) no miocárdio de um rato no grupo ECMI; C) Histologia normal do miocárdio no grupo ECMI+VOR; D) Histologia cardíaca normal no grupo VOR, HE, barras de escala=50 µm.

**Figura 2:**
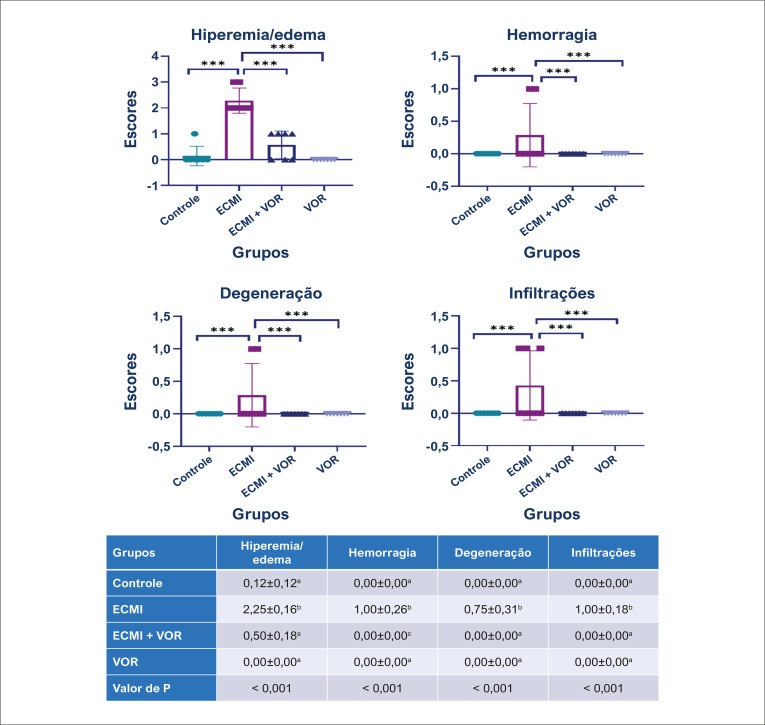
– Resultados da análise estatística dos escores histopatológicos. Os valores são apresentados como médias ± erro padrão. ANOVA de uma via. *** p≤0,001. ECMI: estresse crônico moderado imprevisível; VOR: vortioxetina.

O exame microscópico revelou uma arquitetura de tecido normal no grupo controle. Em contraste, o grupo ECMI exibiu hiperemia, edema, acúmulo de lipídios, degeneração vacuolar, hipereosinofilia no citoplasma de células miocárdicas em alguns ratos e infiltrações de células mononucleares em três ratos. Além disso, uma leve hemorragia foi notada no miocárdio de dois ratos neste grupo. Embora todos os tecidos cardíacos tenham sido examinados, as lesões foram particularmente pronunciadas no ventrículo esquerdo. Ficou evidente que a administração de VOR melhorou esses achados patológicos no grupo ECMI+VOR. Nenhum achado patológico foi observado nos grupos VOR e controle (Figura 1). Os resultados da análise estatística dos achados histopatológicos são apresentados na Figura 2.

### Achados imuno-histoquímicos

**Figura 3:**
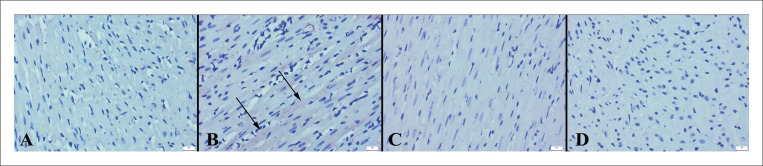
– Expressões de caspase-3 de corações entre os grupos. A) Expressão negativa no grupo controle, B) Aumento da imunoexpressão no miocárdio de um rato no grupo ECMI (setas), C) Expressão negativa no grupo ECMI+VOR, D) Expressão negativa no grupo VOR, método da estreptavidina biotina peroxidase, barras de escala=20µm.

**Figura 4 f05:**
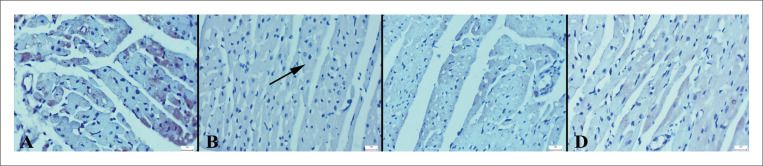
– Expressões de IL-10 de corações entre os grupos. A) Expressão normal no grupo controle, B) Imunorreação diminuída no miocárdio em um rato do grupo ECMI (setas), C) Expressão aumentada no grupo ECMI+VOR, D) Expressão normal no grupo VOR, método da estreptavidina biotina peroxidase, barras de escala=20µm.

**Figura 5 f06:**
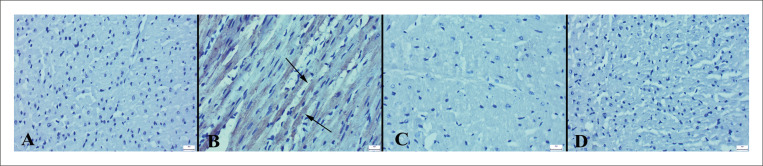
– Expressões de NF-kB de corações entre os grupos. A) Nenhuma expressão no grupo controle, B) Aumento da imunoexpressão no miocárdio no grupo ECMI (setas), C) Expressão negativa no grupo ECMI+VOR, D) Expressão negativa no grupo VOR, método da estreptavidina biotina peroxidase, barras de escala=20µm.

**Figura 6 f07:**
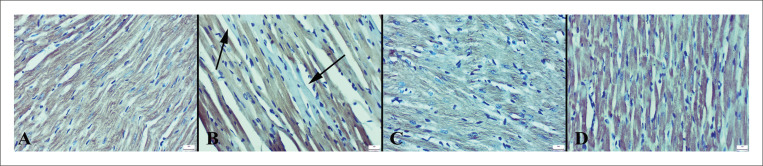
– Expressões de troponina dos corações entre os grupos. A) Expressão significativa no grupo controle, B) Imunoexpressão diminuída no miocárdio no grupo ECMI (setas), C) Expressão significativa no grupo ECMI+VOR, D) Expressão acentuada no grupo VOR, método Streptavidina biotina peroxidase, barras de escala=20µm.

**Figura 7:**
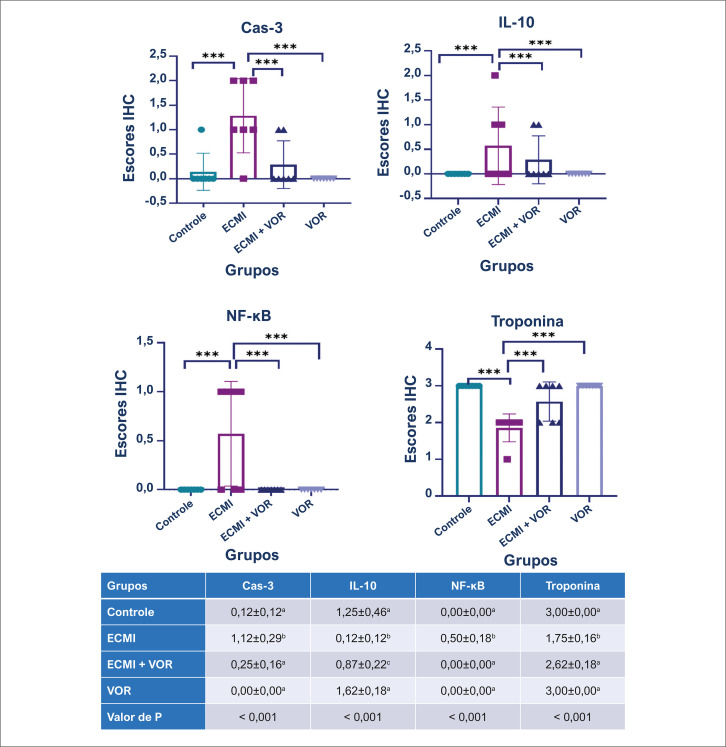
– Resultados da análise estatística dos escores imuno-histoquímicos. Os valores são apresentados como médias ± erro padrão. ANOVA unidirecional. *** p≤0,001. ECMI: estresse crônico moderado imprevisível; VOR: vortioxetina.

Os achados imuno-histoquímicos revelaram um aumento nas expressões de caspase-3 e fator nuclear kappa-light-chain-enhancer de células B ativadas (NF-κB) e uma diminuição nas expressões de IL-10 e troponina no grupo ECMI. No geral, os tratamentos VOR melhoraram as expressões (Figuras 3-6 [Fig f05] [Fig f06] [Fig f07]). A Figura 7 mostra os resultados da análise estatística dos achados imuno-histoquímicos. O possível mecanismo de cafeína e hipotireoidismo no coração é mostrado na Figura Central.

## Discussão

Este estudo demonstrou alterações histopatológicas nos corações de ratos submetidos a ECMI, incluindo hiperemia, edema, micro-hemorragia, acúmulo de lipídios, degeneração vacuolar, hipereosinofilia no citoplasma de células miocárdicas e infiltrações de células mononucleares. Exames imuno-histoquímicos revelaram expressões aumentadas de caspase-3 e NF-κB, indicando apoptose e respostas inflamatórias aumentadas. Ao mesmo tempo, diminuições nas expressões de IL-10 e troponina sugeriram respostas anti-inflamatórias prejudicadas e lesão miocárdica. No entanto, a administração de VOR atenuou significativamente essas alterações patológicas. O tratamento com VOR foi associado a reduções nos níveis de caspase-3 e NF-κB, indicando seu potencial papel protetor contra apoptose e inflamação em tecidos cardíacos. Além disso, VOR contribuiu para a normalização dos níveis de IL-10 e troponina, refletindo respostas anti-inflamatórias e função cardíaca melhoradas. Essas descobertas sugerem que o VOR pode servir como uma abordagem terapêutica promissora para o tratamento da disfunção cardíaca induzida pelo estresse, restaurando o equilíbrio nas vias inflamatórias e apoptóticas.

Estresse, uma preocupação crescente entre indivíduos, pode ser definido como a percepção subjetiva de uma mudança ambiental adversa. Esforços para se adaptar a novas situações geralmente desencadeiam uma resposta significativa ao estresse.^29^ Modelos de ECMI em ratos têm sido amplamente utilizados recentemente para pesquisa sobre o tratamento e patogênese da depressão.^8^ Estudos anteriores relataram que ECMI induz sintomas fisiológicos distintos em animais.^9,10^ Embora existam estudos limitados e recentes examinando os efeitos de ECMI no coração, pouca informação foi publicada sobre os achados histopatológicos e imuno-histoquímicos no coração.^5,30^ No estudo atual, alterações histopatológicas e as expressões de caspase-3, NF-κB, IL-10 e troponina foram investigadas em corações expostos a ECMI em um modelo de rato.

O hipotálamo, a hipófise e as glândulas suprarrenais constituem os principais componentes do eixo hipotálamo-hipófise-adrenal (HPA), envolvendo-se em interações intrincadas relacionadas ao estresse, seja diretamente ou por meio de mecanismos de feedback. Quando o estresse ativa o eixo HPA, ele desencadeia uma secreção aumentada de glicocorticoides adrenais, como o cortisol, pelas glândulas suprarrenais. Os níveis de cortisol plasmático podem aumentar mais de dez vezes em condições intensamente estressantes.^31^ O hormônio liberador de corticotropina (CRH) é um dos hormônios iniciais e cruciais liberados pelo hipotálamo em resposta ao estresse.^32^ Em estudos com animais, a injeção intraventricular de CRH demonstrou induzir comportamentos de resposta ao estresse e elevar a pressão arterial e a frequência cardíaca.^33^ Estudos anteriores relataram que 4 semanas de ECMI aumentaram a frequência cardíaca em repouso e reduziram a variabilidade da frequência em animais experimentais. Além disso, a administração repetida de um novo estressor a animais previamente estressados aumentou as respostas da frequência cardíaca.^22,34^ Em estudos humanos, observou-se que a eliminação de estímulos estressantes restaura as respostas comportamentais, mas as alterações cardiovasculares relatadas persistem.^35^

A seleção de marcadores medidos no coração — caspase-3, NF-κB, IL-10 e troponina — foi baseada em seus papéis estabelecidos em processos fisiopatológicos relacionados ao estresse. A caspase-3 é um efetor-chave na via apoptótica, e sua ativação é indicativa de estresse celular e apoptose em tecidos cardíacos. O NF-κB, um fator crítico de transcrição, está envolvido na resposta inflamatória e é frequentemente ativado sob condições de estresse, contribuindo para a inflamação e remodelação do miocárdio. A IL-10 é uma citocina anti-inflamatória que neutraliza os efeitos de mediadores pró-inflamatórios; sua medição reflete o equilíbrio entre as respostas pró-inflamatórias e anti-inflamatórias no coração. Por fim, a troponina é um biomarcador bem reconhecido para lesão miocárdica e é comumente elevada no soro em eventos cardíacos relacionados ao estresse.^36,37^ Juntos, esses marcadores fornecem uma visão abrangente da interação entre apoptose, inflamação e dano miocárdico, tornando-os adequados para avaliar o impacto do estresse crônico na saúde cardíaca.

Troponina é uma proteína complexa presente nos filamentos finos do aparelho contrátil do miocárdio. Essa proteína facilita a interação entre actina e miosina e desempenha um papel crucial na contração cardíaca.^38,39^ No presente estudo, a expressão da troponina cardíaca foi examinada nos tecidos miocárdicos de ratos submetidos a ECMI. Os achados revelaram uma diminuição nas expressões de troponina em ratos no grupo ECMI, mas o tratamento com VOR melhorou significativamente as expressões de troponina cardíaca no miocárdio. Isso foi interpretado como uma perda de troponina nas células do miocárdio, levando a níveis elevados de troponina no soro enquanto diminui no tecido cardíaco.

Apoptose é um processo de morte celular regulado, ativo e não inflamatório induzido por estímulos fisiológicos ou patológicos. Estudos recentes demonstraram que ECMI afeta o coração,^14,16,40,41^ embora o mecanismo patogênico não esteja totalmente elucidado. A perda de células devido à apoptose em células maduras pode ter consequências graves, e a apoptose pode ser particularmente prejudicial para tecidos como o coração. Em estudos envolvendo pacientes humanos e modelos animais, a ativação de caspases no coração é considerada um marcador de dano.^28,42,43^ No estudo atual, um aumento nas expressões de caspase-3 foi observado apenas no grupo administrado por ECMI, ressaltando os efeitos prejudiciais de ECMI no miocárdio. A apoptose de células miocárdicas desempenha um papel crucial na patologia de doenças cardíacas. Os achados deste estudo revelaram que ECMI aumentou a atividade da caspase-3 em células miocárdicas; no entanto, VOR foi eficaz na redução da atividade apoptótica nessas células em um modelo murino.

Os efeitos relatados da ativação do NF-κB no coração destacam seu papel crucial na regulação da função cardíaca.^44^O miocárdio constitui a maioria da massa cardíaca e do número de células, desempenhando assim o papel mais crucial na função cardíaca.^45^ Além disso, a capacidade de regeneração dos miócitos cardíacos é extremamente limitada, enfatizando a importância do miocárdio para a função normal ao longo da vida.^46,47^ A ativação e a expressão do NF-κB são consideradas marcadores de dano cardíaco.^48^ O aumento significativo nas expressões do NF-κB observado neste estudo indica que o ECMI leva a danos nas células do miocárdio. O VOR foi considerado eficaz na inibição das expressões do NF-κB associadas a danos cardíacos relacionados ao ECMI.

Outros estudos relataram que o estresse psicossocial agudo prolongado leva a um aumento nos marcadores inflamatórios periféricos.^49^ No entanto, há informações limitadas sobre as alterações induzidas pelo estresse em citocinas anti-inflamatórias, como IL-10. O estudo atual demonstrou uma expressão diminuída de IL-10 em células miocárdicas no modelo de rato ECMI. No entanto, o VOR foi eficaz em aumentar as expressões de IL-10.

Com base nos achados histopatológicos, as alterações observadas no tecido cardíaco em nosso estudo sugerem uma ligação potencial entre essas alterações e mecanismos de insuficiência cardíaca ou disfunção miocárdica. A redução nos níveis de troponina e IL-10 pode ser interpretada como indicadores bioquímicos de dano cardíaco, enquanto o aumento na caspase-3 e NF-κB sugere a ativação de processos apoptóticos e inflamatórios. Essas alterações nos biomarcadores indicam o envolvimento de mecanismos celulares que desempenham um papel fundamental na fisiopatologia da insuficiência cardíaca.^50-53^ Numerosos estudos na literatura destacam uma forte relação entre esses biomarcadores e disfunção cardíaca. Embora testes funcionais não tenham sido conduzidos em nosso estudo, nossas descobertas ressaltam a significância clínica desses biomarcadores e estabelecem as bases para investigações futuras mais detalhadas desses mecanismos. Os ajustes necessários para comparações múltiplas nas análises estatísticas e a adição de intervalos de confiança para descobertas importantes fortalecem ainda mais a confiabilidade de nossos resultados.

O NF-κB é responsável pela regulação de genes envolvidos na inflamação e respostas imunes. Foi relatado que o NF-κB desempenha um papel significativo em doenças como doenças cardiovasculares (DCVs), aterosclerose e diabetes. Vários agentes terapêuticos usados para o tratamento de DCVs e diabetes, como pimobendan e inibidores do cotransportador de sódio-glicose 2, exercem efeitos anti-inflamatórios ao inibir a ativação do NF-κB, e foi relatado que a terapia anti-inflamatória tem efeitos benéficos significativos em DCVs.^37^ Também foi relatado que a inibição da via de sinalização do NF-κB reduz os danos cardíacos causados por ECMI.^54^ O aumento significativo nas expressões de NF-κB observado neste estudo indica que o ECMI leva a danos nas células do miocárdio, e nossas descobertas se alinham com as de estudos recentes e novos. Nossos resultados sugerem que o VOR pode ser a melhor escolha para tratar danos cardíacos relacionados ao ECMI.

A DCV é uma fonte primária de morbidade e mortalidade global, tornando crucial entender os mecanismos fisiopatológicos moleculares envolvidos. Recentemente, inúmeras citocinas pró-inflamatórias foram associadas a várias DCVs, que são frequentemente consideradas como representando um estado pró-inflamatório adverso. Entre essas citocinas, as interleucinas e o TNF-α são particularmente proeminentes. A inflamação interage de maneiras complexas com processos fisiopatológicos, como estresse oxidativo e manuseio incorreto de cálcio, e também afeta o equilíbrio entre reparo e destruição de tecidos.^52^ Nesse contexto, evidências pré-clínicas e clínicas demonstraram claramente o papel e a natureza dinâmica das citocinas pró-inflamatórias em muitas condições cardíacas; no entanto, a utilidade clínica dessas descobertas permanece obscura. Portanto, a busca por marcadores que possam revelar a relação entre saúde cardíaca e doença continua. Neste estudo, alterações na troponina, IL-10, caspase-3 e NF-κB relacionadas à lesão cardíaca induzida por estresse foram examinadas imuno-histoquimicamente, e efeitos significativos desses marcadores foram identificados.

O VOR é considerado uma nova abordagem terapêutica no tratamento de pacientes com doença cardíaca, particularmente como um antidepressivo. Pesquisas demonstraram que o VOR possui propriedades anti-inflamatórias e antioxidantes, permitindo que ele reduza os danos aos tecidos cardíacos. Em casos de doença cardíaca isquêmica e insuficiência cardíaca, o VOR é conhecido por proteger os miócitos cardíacos ao inibir a apoptose celular e melhorar a função cardíaca. Além disso, o VOR pode atenuar a produção de citocinas pró-inflamatórias, reduzindo assim a resposta inflamatória no coração. Esses mecanismos sugerem que o VOR pode ser uma opção promissora para o tratamento de doenças cardiovasculares.^53,55-57^ Este estudo também identificou efeitos terapêuticos significativos do VOR em ratos submetidos a ECMI. No entanto, mais estudos clínicos são necessários para estabelecer a eficácia e a segurança do VOR.

A apoptose é uma forma significativa de morte celular em células do miocárdio, e esse processo desempenha um papel crucial na ocorrência de insuficiência cardíaca.^58^ A via da apoptose está intimamente associada à ativação da caspase-3. Estudos anteriores avaliaram várias proteínas de células cardíacas, incluindo mioglobina, troponinas cardíacas T e I (CT-T e CT-I, respectivamente), proteína de ligação de ácidos graxos cardíacos (H-FABP) e proteínas citoesqueléticas, documentando a perda precoce de troponinas cardíacas em lesão miocárdica.^59,60^ Alterações degenerativas agudas, incluindo hemorragia, edema intersticial, necrose de banda contrátil, fibras onduladas, hipereosinofilia citoplasmática, vacuolização perinuclear e alterações vasculares que se manifestam como infiltração de células inflamatórias, foram relatadas como principais lesões cardíacas relacionadas ao estresse em cetáceos encalhados. Além disso, a expressão diminuída da troponina cardíaca foi observada imuno-histoquimicamente em alguns tecidos cardíacos danificados.^61^ Nossos resultados se alinham e apoiam as descobertas do estudo anterior. Particularmente, os resultados deste estudo revelaram um aumento na caspase-3 e uma diminuição nas expressões de troponina nas células miocárdicas de ratos no grupo ECMI. Essas descobertas apoiam a hipótese de que ECMI pode ter efeitos adversos no miocárdio. Além disso, o tratamento com VOR melhorou os achados patológicos relacionados a ECMI.

Embora este estudo ofereça insights valiosos sobre o papel protetor do VOR contra patologias cardíacas induzidas por ECMI, várias limitações devem ser consideradas. Primeiro, o tamanho relativamente pequeno da amostra pode restringir a generalização dos achados, pois coortes maiores podem produzir dados mais robustos e aumentar o poder estatístico da análise. Segundo, o estudo se baseou em um modelo específico de rato, que pode não refletir totalmente a complexidade da fisiopatologia humana associada ao ECMI.

## Conclusões

Embora seja amplamente reconhecido que o estresse psicológico pode ter efeitos adversos nas funções cardiovasculares, os mecanismos precisos subjacentes a esses efeitos ainda não são bem compreendidos. Traços pessoais e atitudes em relação ao estresse podem impactar as respostas fisiopatológicas ao estresse. A complexidade dessas situações representa desafios no desenvolvimento de modelos apropriados de estresse animal e limita a interpretação de experimentos animais a suposições hipotéticas. Em conclusão, este estudo demonstrou mudanças significativas nos achados histopatológicos e imuno-histoquímicos do coração após a exposição ao ECMI no modelo experimental de ratos. Esses resultados fornecem evidências de que o estresse tem efeitos patológicos nas células do miocárdio. Além disso, o tratamento com VOR demonstrou ser eficaz na mitigação desses achados patológicos. Consequentemente, o VOR pode ser a escolha ideal para tratar danos cardíacos em pacientes sob condições de estresse.
