# Supplementary material for: Potencial Terapêutico da Vortioxetina: Respostas Cardíacas ao Estresse Crônico Moderado Imprevisível em um Modelo de Rato
Source: Arq Bras Cardiol. 2025 Feb 5;122(2):e20240159. [Article in Portuguese] doi: 10.36660/abc.20240159 (PMC11805572; doi:10.36660/abc.20240159)
Supplement: Translation

# Translation: Vortioxetine’s Therapeutic Potential: Cardiac Responses to Chronic Unpredictable Mild Stress in a Rat Model

**DOI:** 10.36660/abc.20240159i

**Authors:** Ozlem Ozmen, Serife Tasan, Gulin Ozdamar Unal

**Affiliations:** 1 Türkiye Burdur Mehmet Akif Ersoy University – Department of Pathology, Burdur – Türkiye; 2 Türkiye Suleyman Demirel University – Department of Psychiatry, Isparta – Türkiye

**Keywords:** Vortioxetine, Heart, Immunohistochemistry, Pathology

## Abstract

**Background:**

Stress arises in response to threats or challenges, affecting both physical and mental health. While its harmful effects on the heart are widely recognized, cellular-level investigations remain limited. Antidepressants, including vortioxetine (VOR), are known to impact the cardiovascular system. VOR, used to treat major depressive disorder, is considered a promising option for patients with heart disease due to its anti-inflammatory and antioxidant properties, which may reduce cardiac damage.

**Objectives:**

This study aimed to assess the effects of chronic unpredictable mild stress (CUMS) on rat hearts and evaluate VOR’s potential protective effects against stress-induced cardiac damage.

**Methods:**

Twenty-eight male Wistar Albino rats were divided into four groups. The CUMS group experienced random daily stress for 6 weeks, while the CUMS+VOR group received VOR treatment alongside stress. VOR and control groups were not exposed to stress. Heart samples were examined histopathologically and immunohistochemically.

**Results:**

The CUMS group showed increased hyperemia, hemorrhage, edema, vacuolar degeneration, and mononuclear cell infiltrations, with reduced troponin and IL-10 and increased caspase-3 and NF-κB expressions compared to the control group (p≤0.001). VOR treatment improved these findings, normalizing histopathological and immunohistochemical results.

**Conclusions:**

CUMS caused significant cardiac damage in rats, while VOR treatment showed protective effects by alleviating these pathological changes.

## Introduction

Stress is a significant and escalating psychological issue that impacts the daily lives of individuals globally. Concurrently, depression and anxiety stand out as the most prevalent psychiatric disorders in many societies worldwide.^1^ These psychological disorders emerge from complex interactions involving neurobiological, genetic, and common life event factors.^2,3^

The brain, along with the cardiovascular and immune systems, constitutes common targets of stress. Research indicates that psychosocial factors play a pivotal role in the origin and progression of coronary heart disease. When certain regions of the brain are activated due to stress, the response is generated through the sympathetic nervous system or hormones, consequently influencing the normal functions of the heart. Chronic unpredictable mild stress (CUMS) leads to significant increases in serum corticosterone levels, resulting in an exacerbated atherogenic lipid profile that accelerates atherosclerosis.^4-6^ Gu et al., in particular, observed that CUMS induces morphological and physiological changes that may contribute to atherosclerosis.^7^

The activation of the hypothalamic-pituitary-adrenal axis and the consequent rise in glucocorticoid release, triggered by unpredictable and repetitive stress situations, can result in an amplified production of reactive oxygen species and oxidative stress in the cells of the central nervous system.^4^ Oxidative stress plays a pivotal role in the pathogenesis of cardiovascular system disorders.^8^

CUMS models have been extensively utilized as depression models in experimental animals.^9^ These models replicate social environmental stressors, closely mirroring the development of depression in humans, making them more suitable for investigative purposes.^10-12^ Anhedonic behaviors, characterized by a reduction in sucrose preference in rodents exposed to stress, are key features of this model.^13^ The model’s validity is underscored by the reversibility of this reduction in hedonic effect with chronic treatment involving antidepressant agents.^10^

Grippo et al. reported that rats exposed to chronic mild stress exhibited increased heart rate, reduced heart rate variability, elevated sympathetic cardiac tone, anhedonia, and decreased activity levels in a running wheel. Their findings suggest that rats subjected to CUMS are vulnerable to arrhythmic events, which could lead to further detrimental cardiac outcomes such as myocardial infarction or death.^14^ In addition, it has been reported that it increases heart rate due to chronic stress, contributes to hypertension, and elevates the concentrations of serum cortisol, sodium, and adrenocorticotrophic hormone.^15^ However, there is limited information regarding the histological and immunohistochemical findings in the hearts of rats exposed to CUMS.

The adverse effects of antidepressants on the cardiovascular system have been known for a long time. While antidepressants are generally effective in reducing depression, their use in patients with coronary heart disease remains controversial.^16,17^ Therefore, there is a need to explore antidepressants that do not have harmful effects on the cardiovascular system. Vortioxetine (VOR), a multimodal antidepressant, is employed in the treatment of depression and cognitive disorders associated with depression. VOR treatment has the potential to normalize neurobehavioral changes induced by daily life stressors.^18^ However, the neurobiological mechanisms underpinning the antidepressant and cognitive effects of VOR remain incompletely understood.^19^ VOR is thought to have positive effects on the heart due to its anti-inflammatory and anti-apoptotic properties.

Furthermore, while the impact of stress on coronary diseases is well known, its specific effects on the myocardium remain underexplored. Therefore, the objective of this study is to histopathologically and immunohistochemically examine the damaging effects of CUMS on the heart, with a particular focus on evaluating the protective role of VOR in mitigating CUMS-induced heart lesions in a rat model.

## Materials and Methods

### Animals

In this study, heart tissues were obtained from a project originally focused on investigating the effects of CUMS on brain tissue.^20^ During the histological examination, notable findings were observed in the heart tissues, which prompted further evaluation with the approval of the ethics committee. The study was approved by the Local Ethical Committee on Animal Research at Suleyman Demirel University (Approval No: SDU HADYEK 01/02, dated 06.01.2022). All experiments were conducted in compliance with the ARRIVE guidelines for animal research.^21^

Using GPower 3.1.9.7 software, a study was planned on 4 groups, each consisting of 7 rats (total sample size of 28), considering the relevant parameters (α=0.06, 1-β=0.90, effect size=0.5). In this research, 28 male Wistar Albino rats were acquired from the Suleyman Demirel University Experimental Animal Production and Experimental Research Center, located in Suleyman Demirel University, Türkiye. The rats were 4 weeks old, weighed between 150-200 grams, and were confirmed to be healthy by the supervising veterinarian in the relevant unit. The rats were given a one-week acclimation period before the commencement of the experiment. Following the adaptation period, they were randomly divided (simple random assignment) into four groups, each consisting of seven rats. The control group did not undergo any stress, while CUMS group experienced the chronic unpredictable mild stress procedure. CUMS+VOR group underwent the CUMS procedure and received 10 mg/kg VOR intraperitoneally during the last three weeks of the experiment. The VOR group received 10 mg/kg VOR intraperitoneally during the last three weeks without exposure to stress.

The control and VOR groups were housed in separate rooms from the CUMS and CUMS+VOR groups and were not exposed to any stress. The rats were housed in standard Euro-type 4 cages using wood shavings as litter. All rats were maintained under standard laboratory conditions (temperature: 21°C ± 2°C; humidity: 60% ± 5%; 12/12 h light-dark cycle) and had *ad libitum* access to a standard commercial chow diet (Korkuteli Yem, Antalya, Türkiye) and water, except during the stress periods for the CUMS groups.

### CUMS Model

The CUMS model was implemented based on a previously described method with some modifications.^22,23^ Rats were housed individually and subjected to various stressors as part of the CUMS protocol over 6 weeks. These stressors included 4 hours in a cage tilted at 45°, 24 hours of water deprivation, 24 hours of fasting, continuous illumination, 4 hours in a cage with wet bedding, 4 hours of behavioral restriction, 4 hours of social stress by placing the rats in soiled cages from other rats, 4 hours of water stress in an empty cage with 1 cm of water at the bottom, and 4 hours in an empty cage. A dedicated room in the laboratory was assigned for implementing the CUMS protocol. All stressors were applied individually and continuously, day and night. Control animals were left undisturbed in their cages except for handling during regular cage cleaning.

### Sucrose preference test

Sucrose preference is used as an indicator of anhedonia, one of the key symptoms of depression.^24^ For the sucrose preference test (SPT), animals were given two bottles of 1% w/v sucrose solution and 100 g *ad libitum* on the first day to familiarize them with the taste of sucrose.^25^ On the second day, one of the bottles was replaced with water. On the third day, the rats were deprived of food and water for 23 hours. One bottle contained 100 ml of 1% sucrose solution, while the other held an equal volume of drinking water. The rats were given one hour to choose between the two liquids. To prevent potential preference effects, the positions of the bottles were switched after 30 minutes. The sucrose preference percentage was calculated using the following formula: sucrose preference percentage = sucrose consumption/(sucrose consumption + water consumption).^20^

### Vortioxetine Treatment

Vortioxetine synthesized by H. Lundbeck A/S, Istanbul, Türkiye, was dissolved in distilled water and administered at a dose of 10 mg/kg, as previously outlined.^26,27^ The control and CUMS groups received 0.9% sterile saline. Freshly prepared solutions were intraperitoneally injected at a volume of 1 mL/kg every day at the same time for 3 weeks.^20^ At the conclusion of the experiment, all rats were euthanized under Xylazine HCl (Xylasinbio %2, Bioveta, Czech Republic) + Ketalar HCl (Ketasol, Richter Pharma AG, Austria) anesthesia. Subsequently, samples were collected for histopathological and immunohistochemical analyses.

### Histopathological method

During necropsy, heart samples were collected and preserved in 10% buffered formalin. The tissue samples underwent standard processing using fully automatic tissue processing equipment and were embedded in paraffin wax. Sections of 5 microns in thickness were cut from the paraffin blocks. After drying, the preparations were subjected to alcohol and xylene series, stained with hematoxylin-eosin (HE), mounted with a coverslip, and examined under a light microscope.

A light microscope with a 40x objective was used to evaluate the severity of cardiac lesions, with photos taken from five different regions of each animal’s heart. Lesions were graded based on hyperemia, hemorrhage, edema, lipid accumulation, vacuolar degeneration, hypereosinophilia in the cytoplasm of myocardial cells, and mononuclear cell infiltrations. The severity of histopathological changes was assessed semiquantitatively as follows: 0, normal; 1, mild; 2, moderate; and 3, severe.^28^

### Immunohistochemical examination

Employing the streptavidin-biotin technique following the manufacturer’s instructions, four consecutive sections were mounted on poly-L-lysine-coated slides from previously prepared paraffin blocks and immunohistochemically stained. These sections were utilized for the detection of caspase-3 (Anti-caspase-3 Antibody (E-8): sc-7272, Abcam, UK), IL-10 (IL-10 antibodies, A16445, Bioscience - USA), NF-κB (Anti-NF-κB p65 antibodies (ab16502), Abcam, UK), and cardiac troponin (Anti-cardiac troponin I antibody (FNab09781), FineTest, China) expressions. Each primary antibody was applied at a 1/100 dilution, and sections were incubated with these antibodies for 60 minutes. Immunohistochemistry was performed using biotinylated secondary antibodies and streptavidin-alkaline phosphatase conjugates. The secondary antibody used was the EXPOSURE Mouse and Rabbit Specific HRP/DAB Detection IHC kit (ab80436), and diaminobenzidine (DAB) served as the chromogen (Abcam, Cambridge, UK). Negative controls were treated with an antigen dilution solution instead of the primary antibody. All examinations were conducted on blinded specimens by a specialized pathologist from another university. In the immunohistochemical analysis, each section was independently examined for every antibody. To evaluate the intensity of immunohistochemical reactions in cells marked by these antibodies, a semiquantitative analysis was conducted, employing a grading score ranging from (0) to (3) as follows: (0) negative, (1) focal slight staining, (2) diffuse slight staining and (3) diffuse marked staining. For evaluation, ten different areas under 40X objective magnification in each section were examined. The Database Manual Cell Sens Life Science Imaging Software System (Olympus Co., Tokyo, Japan) was employed for morphometric analyses and microphotography. Results were recorded and statistically analyzed. Immunohistochemical score analyses were carried out using ImageJ version 1.48 (National Institutes of Health, Bethesda, MD).

### Statistical analysis

Statistical analysis was conducted using the Statistical Package for Social Sciences (SPSS) 22.00 (SPSS Inc., Chicago, IL, USA). Initially, the normality of distribution was assessed using the Shapiro-Wilk test. Since the data demonstrated a normal distribution (p>0.05), comparisons between the groups were made with a one-way analysis of variance (ANOVA). The Duncan test was used to identify differences between groups. Values of p<0.05 are considered statistically significant. GraphPad software was used for the graphs.

## Results

### Clinical findings

All rats were observed daily for behavioral changes throughout the study. It was noted that the percentage of sucrose preference significantly decreased in the CUMS group, while the control, CUMS+VOR, and VOR groups exhibited similarly high levels. Additionally, rats in the CUMS group appeared calmer and showed slower movements compared to the other groups. These findings confirm the presence of depressive behaviors in the rats subjected to CUMS.

### Gross findings

At the conclusion of the experimental period, the animals were euthanized in accordance with ethical guidelines, and their hearts were macroscopically examined. No gross pathological changes were identified in the hearts of both the control and study groups during necropsy. The hearts of all rats in the entire group exhibited normal macroscopic appearances.

### Histopathological findings

Microscopic examination revealed a normal tissue architecture in the control group. In contrast, the CUMS group exhibited hyperemia, edema, lipid accumulation, vacuolar degeneration, hypereosinophilia in the cytoplasm of myocardial cells in some rats, and mononuclear cell infiltrations in three rats. Additionally, slight hemorrhage was noted in the myocardium of two rats in this group. Although all cardiac tissues were examined, lesions were particularly pronounced in the left ventricle. It was evident that VOR administration improved these pathological findings in the CUMS+VOR group. No pathological findings were observed in the VOR and control groups (Figure 1). The statistical analysis results of the histopathological findings are presented in Figure 2.

**Figure 1:** – Histopathological appearance of hearts between the groups. A) Normal heart histology in the control group; B) Hyperemia, small hemorrhages (thick arrows), and mononuclear cell infiltrations (thin arrows) in the myocardium of a rat in the CUMS group; C) Normal histology of myocardium in the CUMS+VOR group; D) Normal heart histology in the VOR group, HE, scale bars=50 µm.

**Figure 2:** – Statistical analysis results of the histopathological scores. Values are presented as means ± standard error. One-way ANOVA. *** p≤0.001. CUMS: chronic unpredictable mild stress; VOR: vortioxetine.

### Immunohistochemical findings

Immunohistochemical findings revealed an increase in caspase-3 and nuclear factor kappa-light-chain-enhancer of activated B cells (NF-κB) expressions and a decrease in IL-10 and troponin expressions in the CUMS group. Overall, the VOR treatments improved the expressions (Figures 3 -6 [Fig f05001] [Fig f06001] [Fig f07001]). Figure 7 shows the results of the statistical analysis of the immunohistochemical findings. The possible mechanism of caffeine and hypothyroidism in the heart is shown in the central illustration.

**Figure 3:** – Caspase-3 expressions of hearts among the groups. A) Negative expression in the control group, B) Increased immunoexpression in the myocardium of a rat in the CUMS group (arrows), C) Negative expression in the CUMS+VOR group, D) Negative expression in the VOR group, Streptavidin biotin peroxidase method, scale bars=20µm.

**Figure 4 f05001:** – IL-10 expressions of hearts between the groups. A) Normal expression in the control group, B) Decreased immunoreaction in the myocardium in a rat from the CUMS group (arrows), C) Increased expression in the CUMS+VOR group, D) Normal expression in the VOR group, Streptavidin biotin peroxidase method, scale bars=20µm.

**Figure 5 f06001:** – NF-kB expressions of hearts among the groups. A) No expression in the control group, B) Increased immunoexpression in the myocardium in the CUMS group (arrows), C) Negative expression in the CUMS+VOR group, D) Negative expression in the VOR group, Streptavidin biotin peroxidase method, scale bars=20µm.

**Figure 6 f07001:** – Troponin expressions of hearts between the groups. A) Significant expression in the control group, B) Decreased immunoexpression in the myocardium in the CUMS group (arrows), C) Significant expression in the CUMS+VOR group, D) Marked expression in the VOR group, Streptavidin biotin peroxidase method, scale bars=20µm.

**Figure 7:** – Statistical analysis results of the immunohistochemical scores. Values are presented as means ± standard error. One-way ANOVA. *** p≤0.001. CUMS: chronic unpredictable mild stress; VOR: vortioxetine.

## Discussion

This study demonstrated histopathological changes in the hearts of rats subjected to CUMS, including hyperemia, edema, microhemorrhage, lipid accumulation, vacuolar degeneration, hypereosinophilia in the cytoplasm of myocardial cells, and mononuclear cell infiltrations. Immunohistochemical examinations revealed increased expressions of caspase-3 and NF-κB, indicating heightened apoptosis and inflammatory responses. Concurrently, decreases in IL-10 and troponin expressions suggested impaired anti-inflammatory responses and myocardial injury. However, the administration of VOR significantly mitigated these pathological changes. VOR treatment was associated with reductions in caspase-3 and NF-κB levels, indicating its potential protective role against apoptosis and inflammation in cardiac tissues. Furthermore, VOR contributed to the normalization of IL-10 and troponin levels, reflecting improved anti-inflammatory responses and cardiac function. These findings suggest that VOR may serve as a promising therapeutic approach for managing stress-induced cardiac dysfunction by restoring balance in inflammatory and apoptotic pathways.

Stress, a growing concern among individuals, can be defined as the subjective perception of an adverse environmental change. Efforts to adapt to new situations usually trigger a significant stress response.^29^ Rat models of CUMS have recently been widely utilized for research on the treatment and pathogenesis of depression.^8^ Previous studies have reported that CUMS induces distinct physiological symptoms in animals.^9,10^ Although there are limited and recent studies examining the effects of CUMS on the heart, little information has been published regarding the histopathological and immunohistochemical findings in the heart.^5,30^ In the current study, histopathological changes and the expressions of caspase-3, NF-κB, IL-10, and troponin were investigated in hearts exposed to CUMS in a rat model.

The hypothalamus, pituitary, and adrenal glands constitute major components of the hypothalamic-pituitary-adrenal (HPA) axis, engaging in intricate interactions concerning stress, either directly or through feedback mechanisms. When stress activates the HPA axis, it triggers an increased secretion of adrenal glucocorticoids, such as cortisol, from the adrenal glands. Plasma cortisol levels can surge more than tenfold under intensely stressful conditions.^31^ Corticotropin-releasing hormone (CRH) is one of the initial and crucial hormones released from the hypothalamus in response to stress.^32^ In animal studies, intraventricular injection of CRH has been shown to induce stress-response behaviors and elevate blood pressure and heart rate.^33^ Previous studies have reported that 4 weeks of CUMS increased resting heart rate and reduced rate variability in experimental animals. Furthermore, the repeated administration of a new stressor to previously stressed animals heightened heart rate responses.^22,34^ In human studies, it has been observed that the elimination of stressful stimuli restores behavioral responses, but reported cardiovascular changes persist.^35^

The selection of markers measured in the heart—caspase-3, NF-κB, IL-10, and troponin—was based on their established roles in stress-related pathophysiological processes. Caspase-3 is a key effector in the apoptotic pathway, and its activation is indicative of cellular stress and apoptosis in cardiac tissues. NF-κB, a critical transcription factor, is involved in the inflammatory response and is often activated under stress conditions, contributing to myocardial inflammation and remodeling. IL-10 is an anti-inflammatory cytokine that counteracts the effects of pro-inflammatory mediators; its measurement reflects the balance between pro-inflammatory and anti-inflammatory responses in the heart. Lastly, troponin is a well-recognized biomarker for myocardial injury and is commonly elevated in serum in stress-related cardiac events.^36,37^ Together, these markers provide a comprehensive view of the interplay between apoptosis, inflammation, and myocardial damage, making them suitable for assessing the impact of chronic stress on cardiac health.

Troponin is a complex protein present on the thin filaments of the myocardial contractile apparatus. This protein facilitates the interaction between actin and myosin and plays a crucial role in cardiac contraction.^38,39^ In the present study, cardiac troponin expression was examined in the myocardial tissues of rats subjected to CUMS. The findings revealed a decrease in troponin expressions in rats in the CUMS group, but VOR treatment significantly improved cardiac troponin expressions in the myocardium. This was interpreted as a loss of troponin in myocardial cells, leading to elevated levels of troponin in the serum while decreasing in the heart tissue.

Apoptosis is a regulated, active, non-inflammatory cell death process induced by physiological or pathological stimuli. Recent studies have demonstrated that CUMS affects the heart,^14,16,40,41^ although the pathogenetic mechanism is not fully elucidated. Cell loss due to apoptosis in mature cells can have severe consequences, and apoptosis can be particularly detrimental for tissues like the heart. In studies involving both human patients and animal models, the activation of caspases in the heart is considered a marker of damage.^28,42,43^ In the current study, an increase in caspase-3 expressions was observed only in the CUMS-administered group, underscoring the detrimental effects of CUMS on the myocardium. Myocardial cell apoptosis plays a crucial role in the pathology of heart diseases. The findings of this study revealed that CUMS increased caspase-3 activity in myocardial cells; however, VOR was effective in reducing apoptotic activity in these cells in a murine model.

The reported effects of NF-κB activation on the heart highlight its crucial role in regulating cardiac function.^44^ The myocardium constitutes the majority of heart mass and cell number, thus playing the most crucial role in heart function.^45^ Moreover, the regeneration capacity of cardiac myocytes is extremely limited, emphasizing the importance of the myocardium for normal function throughout life.^46,47^ The activation and expression of NF-κB are considered markers of cardiac damage.^48^ The significant increase in NF-κB expressions observed in this study indicates that CUMS leads to myocardial cell damage. VOR was found to be effective in inhibiting NF-κB expressions associated with CUMS-related cardiac damage.

Other studies have reported that prolonged acute psychosocial stress leads to an increase in peripheral inflammatory markers.^49^ However, there is limited information on stress-induced changes in anti-inflammatory cytokines, such as IL-10. The current study demonstrated a decreased expression of IL-10 in myocardial cells in the CUMS rat model. However, VOR was effective in increasing IL-10 expressions.

Based on the histopathological findings, the observed changes in heart tissue in our study suggest a potential link between these alterations and mechanisms of heart failure or myocardial dysfunction. The reduction in troponin and IL-10 levels can be interpreted as biochemical indicators of cardiac damage, while the increase in caspase-3 and NF-κB suggests the activation of apoptotic and inflammatory processes. These changes in biomarkers indicate the involvement of cellular mechanisms that play a key role in the pathophysiology of heart failure.^50-53^ Numerous studies in the literature highlight a strong relationship between these biomarkers and cardiac dysfunction. Although functional tests were not conducted in our study, our findings underscore the clinical significance of these biomarkers and lay the groundwork for more detailed future investigations of these mechanisms. The necessary adjustments for multiple comparisons in the statistical analyses and the addition of confidence intervals for key findings further strengthen the reliability of our results.

NF-κB is responsible for the regulation of genes involved in inflammation and immune responses. NF-κB has been reported to play a significant role in diseases such as cardiovascular diseases (CVDs), atherosclerosis, and diabetes. Several therapeutic agents used for the treatment of CVDs and diabetes, such as pimobendan and sodium-glucose cotransporter 2 inhibitors, exert anti-inflammatory effects by inhibiting NF-κB activation, and it has been reported that anti-inflammatory therapy has significant beneficial effects in CVDs.^37^ It has also been reported that the inhibition of the NF-κB signaling pathway reduces cardiac damage caused by CUMS.^54^ The significant increase in NF-κB expressions observed in this study indicates that CUMS leads to myocardial cell damage, and our findings align with those of recent and novel studies. Our results suggest that VOR may be the best choice for treating CUMS-related cardiac damage.

CVD is a primary source of global morbidity and mortality, making it crucial to understand the molecular pathophysiological mechanisms involved. Recently, numerous pro-inflammatory cytokines have been linked to various CVDs, which are often considered to represent an adversely pro-inflammatory state. Among these cytokines, interleukins and TNF-α are particularly prominent. Inflammation interacts in complex ways with pathophysiological processes such as oxidative stress and calcium mishandling, and it also affects the balance between tissue repair and destruction.^52^ In this context, preclinical and clinical evidence has clearly demonstrated the role and dynamic nature of pro-inflammatory cytokines in many cardiac conditions; however, the clinical utility of these findings remains unclear. Therefore, the search for markers that can reveal the relationship between heart health and disease continues. In this study, changes in troponin, IL-10, caspase-3, and NF-κB related to stress-induced cardiac injury were examined immunohistochemically, and significant effects of these markers were identified.

VOR is considered a novel therapeutic approach in the treatment of patients with heart disease, particularly as an antidepressant. Research has demonstrated that VOR possesses anti-inflammatory and antioxidant properties, allowing it to reduce damage to cardiac tissues. In cases of ischemic heart disease and heart failure, VOR is noted to protect cardiac myocytes by inhibiting cellular apoptosis and improving heart function. Additionally, VOR can attenuate the production of pro-inflammatory cytokines, thereby reducing the inflammatory response in the heart. These mechanisms suggest that VOR could be a promising option for managing cardiovascular diseases.^53,55-57^ This study has also identified significant therapeutic effects of VOR in rats subjected to CUMS. However, further clinical studies are needed to establish the efficacy and safety of VOR.

Apoptosis is a significant form of cell death in myocardial cells, and this process plays a crucial role in the occurrence of heart failure.^58^ The apoptosis pathway is closely associated with the activation of caspase-3. Previous studies have evaluated various cardiac cell proteins, including myoglobin, cardiac troponins T and I (CT-T and CT-I, respectively), cardiac fatty acid binding protein (H-FABP), and cytoskeletal proteins, documenting early loss of cardiac troponins in myocardial injury.^59,60^ Acute degenerative changes, including hemorrhage, interstitial edema, contractile band necrosis, wavy fibers, cytoplasmic hypereosinophilia, perinuclear vacuolization, and vascular changes manifesting as infiltration of inflammatory cells, have been reported as main stress-related cardiac lesions in stranded cetaceans. Additionally, decreased cardiac troponin expression was immunohistochemically observed in some damaged heart tissues.^61^ Our results align with and support the findings of the previous study. Particularly, the results of this study revealed an increase in caspase-3 and a decrease in troponin expressions in the myocardial cells of rats in the CUMS group. These findings support the hypothesis that CUMS may have adverse effects on the myocardium. Furthermore, VOR treatment improved the CUMS-related pathological findings.

While this study offers valuable insights into the protective role of VOR against cardiac pathologies induced by CUMS, several limitations should be considered. Firstly, the relatively small sample size may restrict the generalizability of the findings, as larger cohorts could yield more robust data and enhance the statistical power of the analysis. Secondly, the study relied on a specific rat model, which may not fully reflect the complexity of human pathophysiology associated with CUMS.

## Conclusions

While it is widely acknowledged that psychological stress may have adverse effects on cardiovascular functions, the precise mechanisms underlying these effects remain not well understood. Personal traits and attitudes towards stress can impact the pathophysiological responses to stress. The complexity of these situations poses challenges in developing appropriate animal stress models and limits the interpretation of animal experiments to hypothetical assumptions. In conclusion, this study demonstrated significant changes in the histopathological and immunohistochemical findings of the heart following CUMS exposure in the experimental rat model. These results provide evidence that stress has pathological effects on the myocardial cells. Moreover, VOR treatment has been demonstrated effective in mitigating these pathological findings. Consequently, VOR may be the optimal choice for treating heart damage in patients under stress conditions.
